# China’s cigarette health warning labels: Undermined by branding

**DOI:** 10.18332/tid/209788

**Published:** 2025-10-10

**Authors:** Qinghua Nian, Katherine C. Smith, Kevin Welding, Jennifer L. Brown, Zhehan Wang, Pinpin Zheng, Chunlin Ren, Joanna E. Cohen

**Affiliations:** 1 Institute for Global Tobacco Control, Department of Health, Behavior and Society, Johns Hopkins Bloomberg School of Public Health, Johns Hopkins University, Baltimore, United States; 2 Department of Preventive Medicine and Health Education, School of Public Health, Institute of Health Communication, Key Lab of Public Health Safety of Ministry of Education, Fudan University, Shanghai, China

**Keywords:** health warning labels, FCTC Article 11, global health, branding, China

## Abstract

**INTRODUCTION:**

In China, health warning labels (HWLs) on cigarette packs are text-only with two messages in rotation. We examined China’s HWLs as they appear on packs, focusing on elements of design and branding that may undermine their effectiveness.

**METHODS:**

We used a systematic protocol to purchase 488 unique cigarette packs in 2023 from five major Chinese cities. The study sample consisted of the 471 packs that displayed the current HWLs mandated by China. A detailed codebook was developed to assess design elements in the HWL area (color/pattern schemes, text to background contrast, branding, etc.) and pack characteristics (brand family). Two trained coders who were native Chinese speakers independently double-coded the packs. We examined the prevalence of identified design elements and compared differences across brand families.

**RESULTS:**

Colors/patterns that appeared on the pack overlapped with the HWL area on almost all packs (90%). About one-quarter (27%) of packs featured branding directly below the HWLs; significant differences were observed across brand families (p<0.05). On 11% of packs, the HWL text lacked contrast against its background. Other concerning design elements included the use of multiple colors in HWL text and background, and split HWLs.

**CONCLUSIONS:**

The prevalence of appealing branding and design elements that overlap the HWL on cigarette packs, potentially diminishes the effectiveness of HWLs and makes the Chinese HWLs less prominent compared to best practices. To enhance the effectiveness of HWLs and align with FCTC Article 11, China could implement stricter HWL regulations prohibiting branding within HWLs.

## INTRODUCTION

In China, the prevalence of cigarette smoking remains a significant public health challenge, with approximately 307.6 million people who smoke, representing nearly one-third of the world’s total smoking population^1^. Effective health warning labels (HWLs) on cigarette packaging can convey health risks associated with smoking and encourage cessation^2^. According to Article 11 of the World Health Organization’s Framework Convention on Tobacco Control (WHO FCTC) requirements, HWLs should cover at least 30% of the principal pack display areas and include color pictures to enhance visibility and impact^3^. The guidelines also include recommendations for the size, rotation, placement, language, and message content.

China ratified the WHO FCTC in 2005, and in 2008 issued regulations to increase HWL coverage to 30% and mandate placement on both the front and back of the pack. Subsequent revisions in 2012 and 2016 changed language from English to Chinese on the back, increased the text size, and expanded HWL coverage to 35%. Despite these changes, China’s HWLs remain text-only, without any requirements for information on smoking-related diseases or cessation support; they are positioned at the bottom of the pack, and are limited to two rotation messages^4,5^. All cigarette packs sold in China – whether they are manufactured by domestic or foreign companies – are legally required to comply with the national tobacco product packaging regulations. The State Tobacco Monopoly Administration (STMA), which plays a key role in China’s implementation of the WHO FCTC, claims that pictorial HWLs are incompatible with Chinese culture^5^.

Despite the fact that more than 90% of packs are compliant with the nation’s HWL requirements vis-à-vis warning location, size, text size, and color contrast^6^, the effectiveness of HWLs in China remains questionable. Among Chinese people who smoke, two-thirds who reported noticing a HWL did not consider quitting^1^. Prior research suggests that China’s HWLs are significantly less effective than the pictorial warnings used in other countries^7,8^. Beyond text-only HWLs and only two messages in rotation, existing regulations do not adequately address design issues that could further compromise HWL effectiveness^9^, including no restriction on branding elements such as colors and patterning in the HWL area. This study examines design issues that potentially undermine public health impact of China’s current cigarette HWLs.

## METHODS

We analyzed data from the Tobacco Pack Surveillance System (TPackSS)^10^, examining 488 unique cigarette packs purchased between 19 April and 23 May 2023 in five Chinese cities (Beijing, Shanghai, Chongqing, Guangzhou, and Kunming). Among those, 471 (97%) packs had the mandated HWLs and were included in this analysis. Cities were selected for their smoking prevalence and diverse geographical locations, with Shanghai being the most populated. In each city, we selected four distinctive neighborhoods within each of three socio-economic strata (low, middle and high) based on property value data. To collect packs, one hub was used as a starting point in each neighborhood. Data collectors followed a standardized protocol^11^, systematically purchasing unique cigarette packs from four types of retailers: independent grocers, tobacco specialty shops, supermarkets, and hypermarkets. A pack was considered unique if it displayed any external variation in its branded design, including differences in pack size, brand name presentation, or color schemes.

Purchased packs were coded for: 1) design colors/patterns on the pack appearing in the HWL area; 2) coder-perceived contrast between HWL text and HWL background; 3) color of HWL text; 4) color of HWL background; 5) branding elements below the HWL; 6) HWL text direction; and 7) placement of HWL. We also coded for brand family of each cigarette pack.

Two trained coders who were native Chinese speakers independently double-coded the packs. All disagreements were discussed and resolved by a senior reviewer who was also a native Chinese speaker. Coders were not instructed related to any *a priori* research questions. Rather, they were instructed only to review the pack photos and record the presence or absence of specific design elements. The inter-rater reliability was high across all codes, with the agreement ranging from 86% to 99%, and prevalence-adjusted and bias-adjusted kappa (PABAK) statistic ranging from 0.72 to 0.97 with an average of 0.89 (Supplementary file Table 1). We used PABAK to provide a more accurate assessment of agreement in the presence of imbalanced prevalence^12^.

### Statistical analysis

We used SAS 9.4 to conduct analyses. Frequencies were used to explore the prevalence of identified design elements. Differences in design elements on HWLs across brand families were examined using Fisher’s exact tests, with p-values estimated via Monte Carlo simulation to account for small cell sizes. Statistical significance was defined as p<0.05 (two-tailed).

## RESULTS

**Figure 1:**
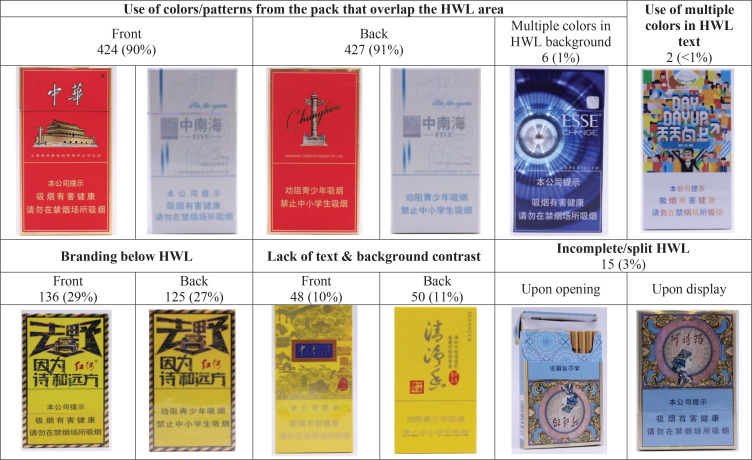
Design elements on cigarette packaging from packs collected in five Chinese cities, TPackSS China, Wave 3, 2023 (N=471)

Among 471 packs that had the required HWLs, the most common compromising design feature was having colors/patterns from the pack that overlapped with the HWL area; this was observed on 90% of the front and 91% of the back of the packs (Figure 1). Additionally, more than one in four packs had branding elements directly below the HWLs, observed in 29% of the front and 27% of the back of the packs. Sufficient contrast between the HWL text and its background was lacking in 11% of packs. We also found instances of multiple colors being used in the HWL text (n=2) and background (n=6). Additionally, some packs (n=15) were designed with the HWL positioned in the flip-top opening area and the branding and design elements printed upside down. As a result, when displayed, the flip-top area appears at the bottom, and upon opening the pack, the HWL on the flip-top becomes split. Significant differences across different brand families were observed in the use of consistent colors/patterns between HWLs and other packaging areas, and in the presence of branding below the HWL (p<0.05) (Supplementary file Table 2).

## DISCUSSION

China’s cigarette packaging often features attractive branding and design elements that potentially detract attention from, and therefore diminish the impact of health messages^13,14^. Beyond the issues of text-only HWLs and only two messages in rotation, this study identifies several design issues in the HWL themselves that are not commonly observed in other countries, including the inclusion of design colors and patterns within HWL areas, HWLs positioned incorrectly, lack of contrast between HWL text and its background, and use of multiple colors in HWL text and background. Such design choices, although compliant with current Chinese regulations, may undermine the potential impact of HWLs and make them less effective compared to best practices recommended by the WHO FCTC.

In China, the effectiveness of HWLs on cigarette packaging may be undermined by regulatory loopholes. Current regulations fail to specify HWL design and placement, allowing prominent pack design colors and decorative elements to be used within and around the HW^15,16^. This enables tobacco companies to employ colors, graphic elements, and packaging designs in and around the HWL that can detract from the regulation’s intended impact. This study also observed differences across brand families in use of branding elements. These differences may arise because, although tobacco companies under China Tobacco – such as Shanghai Tobacco and Guangdong Tobacco – comply with HWL regulatory mandates, they retain some flexibility and control over packaging design of their brands. Local tobacco companies may leverage distinct pack designs to attract customers. Research has consistently shown that the tobacco industry employs tactics such as minimizing warning sizes and introducing novel packaging formats that obscure HWLs and limit impact^17^. These strategies are particularly effective because specific color choices and contrasts can significantly influence consumer perceptions, enhance product appeal and diminish the perceived risks associated with smoking^13,14^. This study underscores the need to strengthen policy by closing regulatory loopholes and enforcing stricter HWL standards in China in line with the WHO FCTC Article 11 recommendations. This will reduce the capacity for cigarette packaging to serve as a promotional tool.

By examining a large and diverse range of cigarette packs, this study provides a broad perspective on HWLs across China. The extensive collection allows for a comprehensive assessment of design elements and compliance with regulations. A detailed analysis of the design elements highlights the nuances in cigarette HWL and packaging design that may negatively affect Chinese people’s perceptions and behaviors regarding smoking.

### Limitations

One limitation of this study is that the study sample reflects a census of unique packs available at retailers we visited, which may not perfectly reflect the packs most frequently purchased by consumers and unpopular brands counted the same as market leaders in this study. We also acknowledge that packs were collected in urban areas of populous cities, and therefore findings on HWL design features and compliance may not be generalizable to less populous or rural areas.

## CONCLUSIONS

The branding and design elements included in and around cigarette HWLs in China could undermine HWL impact by making them less noticeable compared with the more prominent warnings recommended by the WHO FCTC best practices. To improve the efficacy of HWLs and align with the WHO FCTC Article 11 recommendations, it is crucial to implement stricter regulations and close existing loopholes in current regulations. Such measures may help reduce smoking initiation among youth and reduce cigarette consumption and prevalence of current consumers.

## Data Availability

The data supporting this research are available from the authors on reasonable request.
